# Potential and Pitfalls of Mobile Mental Health Apps in Traditional Treatment: An Umbrella Review

**DOI:** 10.3390/jpm12091376

**Published:** 2022-08-25

**Authors:** Jerica Koh, Germaine Y. Q. Tng, Andree Hartanto

**Affiliations:** 1 Millersville University of Pennsylvania, Millersville, PA 17551, USA; 2 Singapore Management University, Singapore 179873, Singapore

**Keywords:** mobile applications, mental health, technology-based care

## Abstract

While the rapid growth of mobile mental health applications has offered an avenue of support unbridled by physical distance, time, and cost, the digitalization of traditional interventions has also triggered doubts surrounding their effectiveness and safety. Given the need for a more comprehensive and up-to-date understanding of mobile mental health apps in traditional treatment, this umbrella review provides a holistic summary of their key potential and pitfalls. A total of 36 reviews published between 2014 and 2022—including systematic reviews, meta-analyses, scoping reviews, and literature reviews—were identified from the Cochrane library, Medline (via PubMed Central), and Scopus databases. The majority of results supported the key potential of apps in helping to (1) provide timely support, (2) ease the costs of mental healthcare, (3) combat stigma in help-seeking, and (4) enhance therapeutic outcomes. Our results also identified common themes of apps’ pitfalls (i.e., challenges faced by app users), including (1) user engagement issues, (2) safety issues in emergencies, (3) privacy and confidentiality breaches, and (4) the utilization of non-evidence-based approaches. We synthesize the potential and pitfalls of mental health apps provided by the reviews and outline critical avenues for future research.

## 1. Introduction

Mobile mental health applications (i.e., apps) are virtual, smartphone-delivered platforms which provide self-directed or remotely facilitated mental health services in the areas of communication, self-monitoring, diagnosis, and treatment [1,2,3]. In order to circumvent user barriers associated with traditional treatment methods—including issues of poor availability, accessibility, and acceptability—these apps offer timely, cost-effective, and discreet channels for users to manage their mental health [3,4,5,6]. Specifically, help-seekers can overcome constraints of traditional clinical settings, such as long waitlists, restricted clinic hours, and living in regions with poor access to mental healthcare [3,4,5,7]. Instead of waiting an average of 14.5 days to consult a clinician [8], relevant information and interventions may be accessed in a timely manner and users may utilize apps for on-demand venting of thoughts and emotions [9,10]. Rather than seeking mental health support in face-to-face settings that require individuals to identify themselves, individuals may access support via apps anonymously and remotely, thus evading negative social evaluation [3,4,5,7].

Critically, as a reflection of the growing demand for mental healthcare [11], mental health apps have undoubtedly seen a rapid increase in their development and adoption. Between 2016 and 2018, they have grown threefold in number [5], offering help-seekers over 10,000 mental health apps to choose from [12]. Further, in a survey of 320 outpatient help-seekers from four clinics in the United States, 70% indicated interest in using apps to facilitate self-monitoring and management of mental health difficulties [13]. Considering their prominence and growing demand, therefore, it is important to inquire into how mental health apps may be utilized in conjunction with traditional interventions.

While an emerging body of research has investigated the utilization of mobile mental health apps in traditional treatment, however, findings have been scant and somewhat polarized. For instance, Torous et al. [14] focused on examination of challenges generated by mental health smartphone apps, while Eisenstadt et al. [15] concentrated on possibilities created by apps. On one hand, several studies have revealed the utility of mental health apps in supplementing different stages of traditional intervention, such as by providing education about treatment techniques prior to enrolment, facilitating symptoms-monitoring during the treatment process, and ensuring continued access to interventions after the treatment period [4,7,16]. On the other hand, a growing body of research has highlighted risks associated with app usage, such as the lack of safeguards around the privacy of users’ information as well as utilization of non-evidence-based approaches [3,5,6]. Given this equivocality, there is a need for a more comprehensive view of the current mobile mental health apps landscape, to guide interested researchers toward a holistic understanding of apps as an adjunct to traditional treatment. As there is an increasing volume of reviews looking into the present mobile mental health landscape, we have chosen to conduct an umbrella review in the hope of presenting a big picture of the evidence base, as well as to discuss congruous or inconsistent findings. An umbrella review is a synthesis of systematic reviews, offering readers opportunities to look at a broad scope of factors investigated by scholars and consider whether consensus in the field has been met. Thus far, past investigations have provided an insightful outline of the current mobile mental health landscape, yet there is a relative lack of umbrella reviews that examined existing overviews. We aim to compile evidence from existing reviews to offer a higher level of summary.

## 2. Methods

### 2.1. Search Strategy and Selection Criteria

We included reviews of mental health apps that reported on: (1) the effectiveness and pitfalls of mobile mental health intervention program(s); and are (2) quantitative or qualitative reviews, rather than individual studies, aimed at reducing subclinical or clinical mental health symptoms. Eligible reviews, up to 31 May 2022, were identified from the Cochrane library, Medline (via PubMed Central), and Scopus databases by two co-authors (J.K., G.T.), using the following search terms: “mental health app *” OR “e%mental health” OR “mobile%based psychotherapy intervention *” OR “app%based mental health intervention *” OR “smartphone%based mental health intervention *” OR “digital mental health” OR “digital app * for mental health” OR “technology in psychotherapy” OR “mental health smartphone app *”) AND (“review*” OR “synthesis” OR “meta-analysis” OR “meta-analytic”).

### 2.2. Quality Assessment

We conducted a methodological quality assessment, using the JBI critical appraisal tool for systematic reviews [17], to evaluate the systematic reviews and meta-analyses included in our umbrella review. This critical appraisal tool comprises eleven items which are rated as “yes”, “no”, “unclear”, or “not applicable”. These include methodological evaluations of each review’s inclusion criteria, search strategy, data synthesis, and strategies to minimize biases in data extraction and study appraisal. For each appraisal item, J.K. and G.T. conducted their evaluations independently and any disagreements were resolved through discussion after independent review. Assessments with at least five “yes” responses were included. In sum, the score (i.e., number of “yes” ratings) of the eligible reviews ranged from a moderate score of five or six (*n* = 4) to a high score of seven and above (*n* = 10). Our quality assessment identified that items four (i.e., “were the sources and resources used to search for studies adequate?”) and six (i.e., “was critical appraisal conducted by two or more reviewers independently?”) had the lowest proportion of “yes” ratings. This highlighted that (1) ensuring a comprehensive search strategy including grey literature; and (2) minimizing bias in critical appraisals are common methodological issues in systematic reviews and meta-analyses. Nevertheless, all fourteen eligible reviews for assessment had at least five “yes” ratings and, therefore, none were excluded from our umbrella review (see Table 1 for critical appraisal results).

### 2.3. Data Extraction

In line with Aromataris et al.’s [31] data extraction protocols for umbrella reviews, the following information was extracted from included reviews: (a) review details (author, year of publication, type of review, review objectives including interventions and outcomes assessed, total sample size, participant demographics, country), (b) search details (number of databases/sources searched, date range of included studies, number of studies included), and (c) analysis details (method of analysis, key findings). The extracted characteristics of included reviews are summarized in Table 2.

### 2.4. Data Synthesis

Due to the heterogeneity of the included reviews in the study aims, mental health interventions, and outcome variables investigated across the included reviews, it was unfeasible to synthesize our results statistically. Instead, we narratively synthesized evidence from various systematic reviews, meta-analyses, scoping reviews, and literature reviews based on the primary findings of each review.

## 3. Results

**Figure 1:**
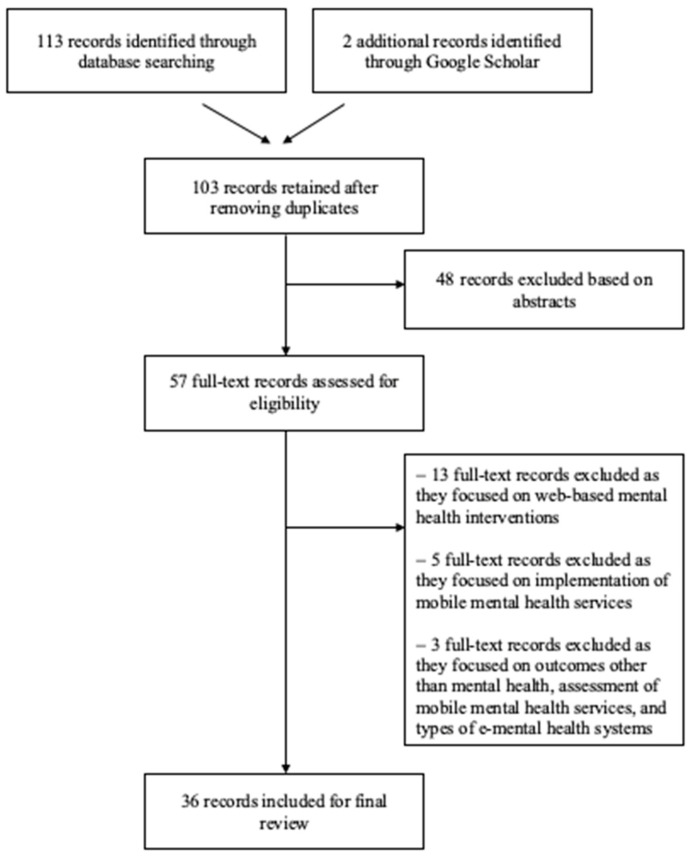
PRISMA diagram of reviews selected for inclusion in the umbrella review.

The main search string returned 103 unique articles (see Figure 1 for PRISMA diagram [51]); and two additional articles were identified via Google Scholar. Thereafter, the article review proceeded in two phases. First, two co-authors reviewed the title and abstract for all 105 articles to determine initial eligibility based on our aforesaid selection criteria, and 48 articles were removed at this phase as they were protocols for literature reviews or articles that did not constitute quantitative or qualitative reviews (e.g., individual studies). In the second phase of article review, the remaining 57 articles were reviewed in full by two co-authors. At this stage, we excluded 13 articles which examined web-based mental health interventions and 5 articles which focused on the implementation of mobile mental health services (e.g., role of therapeutic alliances or gamification elements) [52,53]. In addition, three other reviews were excluded because they focused on outcomes other than mental health (i.e., academic performance [54]), the assessment of mobile mental health services [42], and the types of e-mental health systems and their degree of technological advancement [55]. As a result, a total of 21 articles were excluded at this stage, and 36 articles were included in the final review.

### 3.1. Potential of Mobile Mental Health Apps in Traditional Treatment

#### 3.1.1. Timely Support

In total, 16 out of 36 studies cited timely support as an advantage which mental health apps have provided, by transcending traditional help-seeking boundaries associated with waiting time and physical distance [9,12,15,25,29,33,34,35,36,39,40,42,44,45,49,50]. Given that mental health apps provide in-the-moment support at the user’s convenience, help-seekers can overcome constraints of traditional clinical settings, such as long waitlists, restricted clinic hours, and living in regions with poor access to mental healthcare [8]. For example, Struthers et al.’s [29] systematic review of 24 studies found that time-associated flexibility and level of control over treatment encourage the use of e-mental health services among youths, their parents, and their healthcare providers. Further, Chan and Honey’s [9] integrative review identified that users perceive mobile mental health apps as being “easy to use”, since app usage may be accessed on demand and can be easily integrated into the user’s daily routines. Considering that delayed treatment contributes to more severe and enduring mental health difficulties [4,8,56], the timely nature of mobile mental healthcare is especially helpful in situations when an in-the-moment experience of relief is needed and traditional support might not be as helpful by the time it becomes available [33].

#### 3.1.2. Cost-Effective

Further, as cited in 11 reviews, mental health apps afford users with the opportunity to access cost-effective treatment options according to their financial abilities [25,32,34,35,36,42,44,46,48,49,50]. For instance, Binhadyan et al.’s [35] literature review of 74 articles—which addressed e-mental health interventions for university students with ADHD—identified that the minimal (or no) fees for app-based interventions played a key role in enabling help-seekers to circumvent barriers to traditional mental healthcare. Echoing this, Oyebode et al.’s [46] thematic analyses of user reviews of 106 mental health apps found that the average price of 11 fee-based apps was USD 5.26, which is significantly lower than average psychotherapy fees ranging from USD 100 to USD 200 per session in the United States [57]. Hence, the lower cost of digital apps, compared to traditional psychotherapy, renders mental health apps a more accessible psychological tool for people of varying financial abilities.

#### 3.1.3. Combat Stigma in Help-Seeking

Notably, eight studies noted that mental health apps provide the ability to access mental healthcare discreetly and thus circumvent the adverse stigma surrounding help-seeking [15,35,36,39,44,48,49]. For example, in Lal and Adair’s [44] literature review of 115 articles about e-mental health interventions, it was highlighted that digital mental health interventions allow individuals who are uncomfortable with in-person treatment to receive help anonymously and bypass discomforts associated with identifying themselves and facing negative social evaluations. This may be especially helpful for people from collectivist cultures with prevalent “face” concerns, where conventional help-seeking has been found to be associated with poorer life satisfaction and lower positive affect [58]. Moreover, Wies et al.’s [50] review of 26 digital mental health treatments revealed that apps could serve as an initial point of contact and gradually facilitate transition to face-to-face interventions. In sum, mobile mental health apps potentially allow their users to overcome help-seeking barriers stemming from stigmatized attitudes toward conventional mental healthcare.

#### 3.1.4. Enhance Therapeutic Outcomes

As highlighted in 25 studies, mobile mental health apps may also enhance therapeutic outcomes (see Table 3 for a summary of the target populations of the included reviews) including reducing symptoms of mood disorders [9,12,15,19,20,22,23,24,25,27,28,29,30,35,36,37,38,40,41,42,46,47,48,49,50]. For instance, in Firth et al.’s [19] meta-analysis of 18 randomized controlled trials, smartphone interventions had a small-to-moderate effect in reducing depressive symptoms in an overall sample of 3, 414 adults from both clinical and nonclinical populations. Further, Petrovic and Gaggioli’s [47] review of eight studies on mobile-based mental health tools showed that participants experienced reduced stress levels and improved coping skills after three weeks of app usage, suggesting that apps increase the likelihood of treatment success by providing opportunities to practice coping strategies in clients’ natural environments. Harith et al.’s [41] umbrella review of seven studies also found significant evidence of the effectiveness of digital mental health interventions, including app-based programs, in alleviating depression, anxiety, stress, and eating disorder symptoms in university students.

More specifically, mental health apps can amplify treatment outcomes by complementing different stages of traditional interventions in line with their specific purpose. For example, in Hwang et al.’s [42] scoping review, certain mental health apps (e.g., MoodPrism, mHealth)—which track and monitor users’ emotional state and psychological stress—were found to reduce symptoms of depression, anxiety, and stress. Hence, by providing on-the-go documentation of users’ psychological well-being, these apps can tailor relevant goals for each user in real-time and supplement traditional treatment. In addition, Oyebode et al.’s [46] thematic analysis of user reviews of 104 mental health apps revealed positive themes such as “reminder and notification”, “in-app support”, “logging”, “analytics and visualization”, “assessment”, and “data export”; which indicate the unique features of mental health apps that are valued by help-seekers. Together, this suggests that users could utilize mental health apps in conjunction with traditional treatment to enjoy higher therapeutic success as compared to only receiving the traditional face-to-face intervention alone. Nonetheless, common themes for the pitfalls of mental health apps have been identified as well.

### 3.2. Pitfalls of Mobile Mental Health Apps in Traditional Treatment

#### 3.2.1. User Engagement Challenges

Six reviews referred to high attrition rates and poor rates of sustained engagement prevalent among mental health apps [14,20,22,24,29,30]. For instance, Garrido et al.’s [20] review of 32 digital mental health interventions found that 39% of studies reported attrition rates of over 20%—levels indicative of potential attrition bias. Further, in Struthers et al.’s [29] review of 24 studies on acceptability of e-mental health for youths, the number of participants who completed the full intervention ranged widely across studies from 29.4% to 87.5%, with two studies suggesting the decreasing usage of e-mental health interventions over time. As theorized by Torous et al. [14], user engagement may be hindered by factors including unsatisfactory functionality of these apps and usability concerns (i.e., difficulties using apps).

#### 3.2.2. Safety Issues in Case of Emergency

According to two reviews [14,21], mental health apps may also be poorly equipped to assist users through emergencies. For instance, Larsen et al. [21] reviewed publicly available apps which address suicide, and found that none of these apps abided with the best practice of providing visible crisis support information within the app. Similarly, in Torous et al.’s [14] clinical review of challenges surrounding user engagement, it was suggested that the vast majority of apps are limited in their ability to respond effectively during emergencies related to suicide or self-harm, or recognize anticipatory warning signs. In the event of a time-sensitive mental health emergency such as risk of suicide, therefore, help-seekers might not be able to access critical support needed through mental health apps).

#### 3.2.3. Confidentiality Breaches

In 12 out of 36 included reviews, privacy and confidentiality breaches were consistently cited as a key concern among mental health app users, with these concerns falling into two categories: (1) third-party access to confidential information; and (2) lack of an explicit privacy policy [9,14,18,21,26,29,32,39,45,46,49,50].

First, eight reviews found that mental health app users were commonly concerned with their confidential information being shared with third parties or used for unauthorized purposes such as marketing [9,14,18,29,39,45,46,50]. In Wies et al.’s [50] scoping review of ethical challenges in digital mental health, it was shown that mental health app users’ main concerns centered around the consequences of confidential information being leaked to third parties, which would implicate professional, personal, and social domains of their lives. In particular, two reviews identified inadequate passcode protection (i.e., to prevent external access to users’ data) as a privacy-related weakness of mental health apps [32,49]. For instance, a thematic analysis of user reviews of 106 mobile mental health apps revealed that mental health app users were dissatisfied with the lack of passcode protection (e.g., a unique PIN) to prevent external access to sensitive information [32].

A second concern was the lack of clear privacy policies which explain the protection of users’ information, as highlighted by five reviews [21,26,32,46,49]. More specifically, only 22% of apps targeted at bipolar disorder and 29% of apps targeted at suicide or deliberate self-harm provided a clear privacy policy which informs users on how their data are used [21,26]. Moreover, Wies et al. [50] reported that there is insufficient clarity about the adequacy of consent obtained through digital mental health apps, in particular regarding the type of data processing or intervention that the user is consenting to. Taken together, therefore, the use of mental health apps is often accompanied by risks of being identified as a help-seeker or the leakage of personal information to third parties, thus endangering users’ privacy and impeding trust and engagement with these apps.

#### 3.2.4. Utilization of Non-Evidence-Based Approaches

Lastly, limited empirical and theoretical evidence has been found for both (1) the efficacy of mental health apps and (2) the basis of therapeutic interventions used in mental health apps.

First, 10 reviews found limited evidence for the effectiveness of mental health apps in reducing symptoms of psychological distress (e.g., depression, anxiety, stress) and improving socioemotional competency [14,23,24,36,38,40,42,45,47,50]. For example, Drissi et al.’s [38] systematic review of studies examining e-mental health interventions developed for healthcare workers found that only two studies (27%) included empirical evaluations of the reported interventions, and the empirical evaluations were based on a limited number of participants. Similarly, Gould et al.’s [40] review of mental-health-related apps created by the Veteran Affairs or the Department of Defense showed a pressing lack of evidence for the effectiveness of these apps, with the exception of two apps (PTSD Coach, Virtual Hope Box). Further, in studies examining the efficacy of app interventions, there has been a lack of empirical support for their long-term effectiveness. In Carter et al.’s [36] review of 37 digital mental health intervention studies, for instance, 23 studies (62%) reported results from less than 6-months follow-up. In addition, in Leech et al.’s [23] systematic review of mental health apps for adolescents and young adults, all 11 randomized controlled trials examined the immediate or short-term effects of app interventions, except four studies which incorporated 6-week to 6-month follow-up assessments. Together, this suggests that the long-term benefits of mental health app usage have not been established by empirical evidence, and help-seekers should not rely entirely on these platforms for mental health treatment.

Second, apart from the efficacy of mobile mental health apps, four reviews cited an insufficient theoretical and empirical basis for therapeutic techniques employed by mental health apps [14,42,47,50]. For example, Petrovic and Gaggioli [47] conducted a scoping review of digital mental health tools catered to informal caregivers in Europe, and found that only a small portion of their 16 reviewed papers defined a clear therapeutic rationale behind the interventions used, such as adopting principles of cognitive behavioral therapy or stress inoculation training. In addition, Hwang et al. [42] conducted a scoping review of 14 studies about mental-health-related apps for adults over 18 years of age, and identified two studies that did not provide theoretical evidence for their intervention methods, involving a breathing exercise app and mood-monitoring app. Seeing as such unsupported practices could unintentionally pose serious risks to the well-being of help-seekers in dangerous situations, it is crucial that clinicians and researchers remain astute as to the scientific evidence informing app-based mental healthcare.

## 4. Discussion and Conclusions

### 4.1. Key Findings

**Figure 2:**
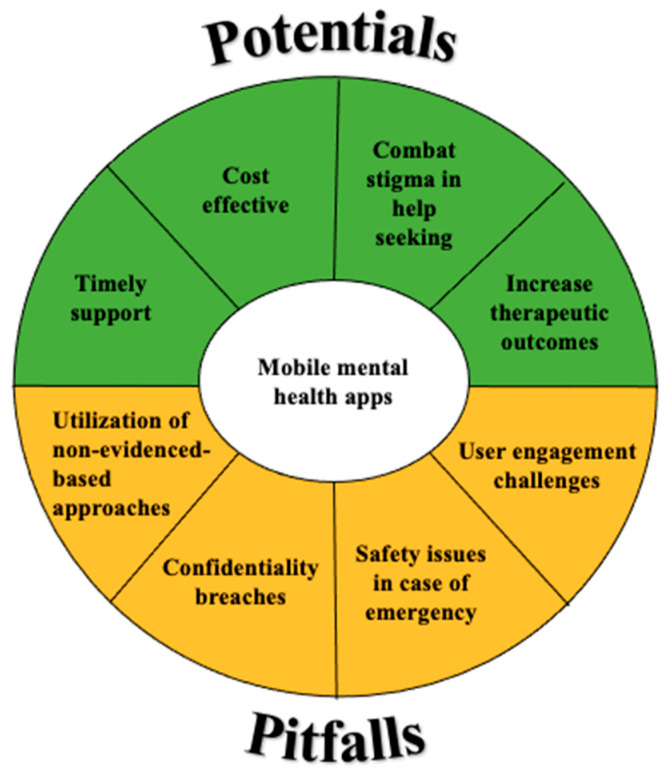
Potential and pitfalls of mobile mental health apps.

In sum, mobile mental health apps can potentially circumvent barriers of traditional mental healthcare to provide timely, cost-effective, and discreet support which facilitates various stages of treatment and improves outcomes. On the other hand, it is imperative that app users (clinicians and help-seekers) are mindful of the pitfalls surrounding apps usage: these involve engagement challenges, safety issues, confidentiality breaches, and a lack of evidence-based practices (see Figure 2 for an overview).

### 4.2. Strengths and Limitations

Our review has several limitations that should be noted. First, given that our umbrella review provided a higher-level synthesis of a wide range of previous reviews, this introduced significant heterogeneity—regarding review methodologies (e.g., systematic reviews, narrative reviews, thematic analyses), primary focus of the reviews (e.g., efficacy, user engagement, ethical challenges), sample demographics (e.g., adolescents, caregivers, young adults), and outcome measures used (e.g., posttraumatic stress symptoms, depression symptoms, emotion regulation)—hence introducing difficulties with interpretation of common potential and pitfalls of mobile mental health apps. Nonetheless, this cross-review heterogeneity reinforces the need for the present umbrella review which identifies converging themes of mental health apps’ advantages and downfalls despite varying aims and measures. Second, since we included only published peer-reviewed reviews written in English, unpublished work and reviews in other language mediums were not included in our search strategy; hence, this may have influenced the findings of this review. Further, as we searched three key databases, our search strategy may have excluded relevant reviews from other databases such as PsycINFO and EMBASE. Our inclusion criteria for reviews may have resulted in overlap of primary studies between reviews. Finally, due to the rapidly advancing nature of digital mental health interventions, it is possible that some of the mobile mental health apps assessed may now be outdated. In spite of these limitations, strengths of the present umbrella review include its strict adherence to methodology protocols for umbrella reviews (e.g., utilization of JBI critical appraisal checklist), holistic synthesis of evidence for both potential and pitfalls of mobile mental health applications, and inclusion of a broad evidence base including systematic reviews, meta-analyses, scoping reviews, and literature reviews.

### 4.3. Future Research Directions

To support the continued examination of app usage as an adjunct to traditional treatment, future research could inquire into three key areas.

#### 4.3.1. App Functions

First, in terms of app *functions*, further research should examine the efficacy of mental health apps in supporting individuals with differing degrees of symptom severity. Considering that mental health apps are commonly designed and utilized to manage and relieve mild symptomatology [59], there is currently a lack of understanding regarding how these approaches may be applied to more severe symptoms. As app effectiveness may vary across the mild, moderate, and severe ranges of mental health conditions, future investigations could probe into how people with different levels of symptom severity (e.g., depression severity) respond to symptom relief provided by mental health apps.

#### 4.3.2. App Regulation

Second, regarding app *regulation*, there is a need for further research to develop overarching evaluation guidelines for mental health apps. Due to the present lack of such guidelines, standardized criteria for “approved-for-use” apps remain unclear to both app developers and clinicians alike [5,60]. Hence, future studies should examine key elements for the regulation of mental health apps, such as the presence of evidenced-based approaches, existing randomized controlled trials conducted to assess app efficacy, as well as visibility of emergency services contacts. In so doing, both app developers and mental health professionals may achieve a shared understanding of the key elements guiding evaluation and regulation of mental health apps.

#### 4.3.3. Individual Differences in Apps Usage

Finally, with regard to *individual differences* in apps usage, future research should look into the role of individual differences—including demographic factors and individual needs and preferences—in modulating the effectiveness of mental health apps [61,62]. Research has suggested that trait-like demographic and usage factors, including socioeconomic background, individual motivations underlying digital technology use, perceptions of usefulness, and smartphone use preferences could potentially influence access to and well-being outcomes of digital technology, including mental health apps [5,62,63]. Given that the potential of mental health apps has primarily been examined in adolescents and young adults (see [23] for a review), however, there is currently a lack of understanding about the role of these individual differences, such as demographics (e.g., socioeconomic status, age) and other usage factors, in shaping engagement with and effectiveness of mental health apps. Therefore, subsequent research should inspect how these individual difference factors influence apps effectiveness.

### 4.4. Conclusions

In sum, this umbrella review provided a comprehensive synthesis of existing quantitative and qualitative evidence regarding the potential and pitfalls of mobile mental health apps as an adjunct to traditional psychotherapy. Further, we offer three key areas for future research, concerning app functionality, app regulation, and individual differences in app usage. Our review highlights that mobile mental health apps’ unique potential, such as providing timely support, being cost-effective, combating stigma surrounding help-seeking, and enhancing treatment outcomes, could be tapped into to supplement mental health interventions, although associated risks (i.e., user engagement challenges, safety issues, confidentiality breaches, and non-evidence-based approaches) need to be understood and managed. Specifically, one viable risk management strategy would be adhering to the American Psychiatric Association’s hierarchal framework that emphasizes clinicians’ responsibilities to examine stages of the framework with clients, discuss queries, and support shared decision-making on app usage [61].

## Figures and Tables

**Table 1:** JBI critical appraisal of systematic reviews.

**Question ^1^**	**Systematic Review/Meta-Analysis**						
	**Borghouts et al. [18]**	**Eisenstadt et al. [15]**	**Firth et al. [19]**	**Garrido et al. [20]**	**Larsen et al. [21]**	**Lattie et al. [22]**	**Leech et al. [23]**
1. Is the review question clearly and explicitly stated?	Unclear	Yes	Yes	Yes	Yes	Yes	Yes
2. Were the inclusion criteria appropriate for the review question?	Yes	Yes	Yes	Yes	Yes	Yes	Yes
3. Was the search strategy appropriate?	Yes	Yes	Yes	Yes	Yes	Yes	Yes
4. Were the sources and resources used for the study adequate?	Unclear	Unclear	Yes	Unclear	Yes	Unclear	Yes
5. Were the criteria for appraising studies appropriate?	Yes	Yes	NA	Yes	NA	Yes	Yes
6. Was critical appraisal conducted by two or more reviewers independently?	No	Yes	NA	No	NA	Yes	No
7. Were there methods to minimize errors in data extraction?	No	Yes	No	Yes	Unclear	Yes	No
8. Were the methods used to combine studies appropriate?	Yes	Yes	Yes	Yes	Yes	Yes	Yes
9. Was the likelihood of publication bias assessed?	NA	NA	Yes	No	NA	NA	NA
10. Were recommendations for policy and/or practice supported by the reported data?	Yes	Yes	NA	Yes	Yes	NA	NA
11. Were the specific directives for new research appropriate?	Yes	Yes	Yes	Yes	Unclear	Yes	Yes
Overall appraisal ^2^	Incl.	Incl.	Incl.	Incl.	Incl.	Incl.	Incl.
**Question ^1^**	**Systematic Review/Meta-Analysis**						
	**Lehtimaki et al. [24]**	**Liverpool et al. [25]**	**Nicholas et al. [26]**	**Simblett et al. [27]**	**Six et al. [28]**	**Struthers et al. [29]**	**Zhang et al. [30]**
1. Is the review question clearly and explicitly stated?	Yes	Yes	Yes	Unclear	Yes	Yes	Yes
2. Were the inclusion criteria appropriate for the review question?	Yes	Yes	Yes	Yes	Yes	Yes	Yes
3. Was the search strategy appropriate?	Yes	Yes	Yes	Yes	Yes	No	Yes
4. Were the sources and resources used for the study adequate?	Unclear	Unclear	Yes	Unclear	Unclear	Yes	Unclear
5. Were the criteria for appraising studies appropriate?	Yes	Yes	NA	Yes	Yes	Yes	Yes
6. Was critical appraisal conducted by two or more reviewers independently?	Yes	Yes	NA	No	No	Yes	Yes
7. Were there methods to minimize errors in data extraction?	Yes	Yes	Unclear	No	Yes	Yes	Yes
8. Were the methods used to combine studies appropriate?	Yes	Yes	Yes	Unclear	Yes	Yes	Yes
9. Was the likelihood of publication bias assessed?	NA	NA	NA	No	Yes	NA	NA
10. Were recommendations for policy and/or practice supported by the reported data?	Yes	Yes	Yes	Yes	Yes	Yes	Yes
11. Were the specific directives for new research appropriate?	Yes	Yes	Unclear	Yes	Yes	Yes	Yes
Overall appraisal ^2^	Incl.	Incl.	Incl.	Incl.	Incl.	Incl.	Incl.

^1^ Possible responses: yes/no/unclear/not applicable. ^2^ Possible responses: include/exclude/seek further information.

**Table 2:** Characteristics of included reviews.

Authors, Year of Publication	Review Type	Review Objective	Total Sample Size	Participant Demographics (Age)	Country	Number of Sources Searched	Date (Year) Range of Included Studies	Number of Studies Included	Method of Analysis	Key Findings
Alqahtani and Orji [32]	User review analysis	Examine strengths and weaknesses of apps	13,549 user reviews	NA	NA	2 (Apple’s app store, Google Play)	NA	106 mental health apps	Thematic analysis	Apps interface and user-friendliness are strengths. Apps lack content, personalization, security, and privacy.
Bakker et al. [33]	Lit. review	Provide recommendations for future apps development	27 mental health apps	NA	NA	3 (PsycInfo, Scopus, ProQuest)	Mar 1975–Mar 2015	27 mental health apps	NA	Current lack of trial-based evidence for apps, need more RCTs.
Balcombe et al. [34]	Lit. review	Summarize and evaluate digital mental health for athletes	NA	NA	NA	2 (PubMed Central, Directory of Open Access Journals)	2016–2020	NA	Systematic review	Apps’ real-time function helpful for symptom tracking and mental health screening. Apps face engagement issues.
Binhadyan et al. [35]	Lit. review	Examine current trend of e-mental health and issues related with ADHD	NA	16 and above	NA	7 (ACM Digital Library, ScienceDirect, IEEE Eplore, SpringerLink, ProQuest, Australian Standards, Google Scholar)	After 2004	74	Systematic review	E-mental health improves treatment accessibility, reduces cost, and enhances quality.
Borghouts et al. [18]	Systematic review	Identify barriers and facilitators affecting e-mental health user engagement	NA	16 and above	NA	5 (SCOPUS, PubMed, PsycINFO, Web of Science, Cochrane Library)	After 2010	208 articles	Systematic review	Barriers: severe mental health, apps have technical and lack of personalization issues. Facilitators: social connections and mental health awareness.
Carter et al. [36]	Lit. review	Provide benefits of digital mental health interventions in low-middle and middle income countries	NA	NA	East, Central, South Asia, Central Latin America, Middle East, Eastern Europe, Southeast Asia, Africa	1 (Medline)	2016–2020	37 articles	Systematic review	Digital mental health can help to detect, diagnose, prevent, and treat mental health disorders in these countries.
Chan and Honey [9]	Lit. review	Identify users perception of mental health apps	NA	18 and above with mental health condition	USA, Spain, Sweden, UK, Europe, Asia, Dominican Republic, Canada, Germany, Australia	4 (CINAHL, Embase, Medline, PsycInfo)	After 2000	17 articles	Integrative review	Apps are useful supplement to treatment. Ease of use, content, and privacy are concerns of apps usage.
Denecke et al. [37]	Lit. review	Identify aspects of CBT in mental health apps	NA	18 and above	NA	3 (PubMed, IEEE Xplore, ACM digital library)	2007–2020	34 articles	Narrative synthesis	Promote self-monitoring and self-management strategies.
Drissi et al. [38]	Lit. review	Identify e-mental health for healthcare staff	NA	NA	China, UK, Iran, Canada, USA, Malaysia	5 (IEEE, ACM, ScienceDirect, Scopus, PubMed)	2020 onwards	11 articles	Systematic review	E-mental health helpful but lacks empirical evidence.
Eisenstadt et al. [15]	Systematic review and meta-analysis	Identify features of mental health apps and evaluate potential	48 mental health apps	18–45	15 countries	5 (Medline, Embase, PsycInfo, Web of Science, Cochrane Central)	Up to 2021	52 articles	Narrative synthesis and meta-analysis	Apps promote emotion regulation, mental health, and well-being.
Ellis et al. [39]	Scoping review	Assess e-mental health gaps in relation to COVID-19.	NA	NA	USA, Australia, Canada, UK, India	4 (Medline, Embase, PsycInfo, CINAHL)	2019–2021	356 articles	Narrative techniques	Privacy and safety regulations, lack integration into healthcare models and accountability framework.
Firth et al. [19]	Meta-analysis	Examine efficacy of using smartphones for treatment of depression	3414	18–59.3	NA	7 (Cochrane Central, Health Technology Assessment Database, AMED, HMIC, Ovid Medline, Embase, PsycInfo)	Until 2017	18 RCTs	Comprehensive Meta-analysis 2.0	Smartphones are a promising self-management tool for depression.
Garrido et al. [20]	Systematic review and meta-analysis	Examine effectiveness of digital mental health for anxiety and depression in young people	NA	12–25	Australia, USA, Asia, North Europe, South America	4 (PsycInfo, PubMed, ProQuest, Web of Science)	2007–2017	41 articles	Thematic analysis and narrative analysis	Treatment effect high when supervision was present, content and interface important to users.
Gould et al. [40]	Lit. review	Summarize feasibility, usability, efficacy, effectiveness of mental health apps	NA	NA	NA	1 (EBSCOhost)	Until 2018	22 articles	Systematic review	There is evidence for feasibility and acceptability, research for efficacy and effectiveness is scarce.
Harith et al. [41]	Umbrella review	Synthesize and evaluate digital interventions targeting university students	NA	University students	Australia, UK, USA, Canada, Norway, Spain, China, Europe	5 (PubMed, Psychology and Behavioral Science Collection, Web of Science, ERIC, Scopus)	2000–2021	7 articles	Narrative synthesis	Digital interventions were effective; effectiveness depended on delivery format, mental health condition, and population.
Henson et al. [12]	Lit. review	Determine digital therapeutic alliance in smartphone interventions for mental illnesses	NA	17–65	NA	4 (PubMed, PsycInfo, Embase, Web of Science)	2018 onwards	5 articles	Systematic review	Smartphones enhance therapy engagement and adherence, therapeutic alliance in allowing communication outside therapy hours was key.
Hwang et al. [42]	Scoping review	Examine effects of mobile mental health apps for adults	NA	18 and above	NA	8 (RISS, DBpia, Medline, CINAHL, Embase, PsycInfo, Cochrane Library, Google Scholar)	2010–2019	14 articles	Systematic review	Apps based on theoretical knowledge and empirical evidence were lacking.
Kaveladze et al. [43]	Secondary data analysis	Examine relationship among subjective user experience and objective measures of apps popularity and engagement	NA	NA	NA	4 (MARS, Apple App Store, Google Play, MAU)	2020–2021	56 apps	Statistical analyses using R	User experience does not predict sustained engagement with apps. Need to understand the link between user experience and engagement.
Lal and Adair [44]	Rapid literature review	Review the literature on e-mental health, including its applications, strengths, limitations, and evidence base	NA	NA	USA, Australia, the Netherlands	1 (MEDLINE)	2000–2010	115	Descriptive review	E-mental health applications address information provision; screening, assessment, and monitoring; intervention; and social support.
Larsen et al. [21]	Systematic overview	Compare evidence-based strategies undertaken for suicide prevention with the content of publicly available apps providing tools for suicide prevention	NA	NA	NA	2 (Australian Google Play store, Australian iTunes store)	NA	123 apps	Systematic review	Strongest evidence of suicide prevention strategies found for facilitating access to crisis support. All reviewed apps employed at least one strategy that aligned with best-practice or evidence-based guidelines.
Lattie et al. [22]	Systematic review	Identify the effectiveness, usability, acceptability, uptake, and adoption of digital mental health interventions focused on depression, anxiety, and enhancement of psychological well-being among college students	NA	NA	Mexico, Canada, USA	5 (MEDLINE, EMBASE, PsycINFO, Web of Science, and the Cochrane Library)	Up to 2019	89	Systematic review	The majority of programs were effective or partially effective in producing beneficial changes in the main psychological outcome variables.
Leech et al. [23]	Systematic review	Provide a systematic, quantitative review of current research to address whether app-based interventions are effective in managing adolescents and young adults’ mental health symptoms compared to wait-list controls or another comparison condition.	1706	Mostly adolescent females (65% female; Mean age = 18.9 years, SD = 3.5)	Australia, UK, USA	4 (Embase, Cochrane Library, PsycINFO, PubMed)	2011–2020	11 RCTs	Meta-analysis	App interventions produced significant symptom (depression, stress) improvement across multiple outcomes, compared to wait-list or attention control conditions.
Lehtimaki et al. [24]	Systematic overview	Synthesize the current evidence on digital health interventions targeting adolescents and young people (aged 10–24 years) with mental health conditions, with a focus on effectiveness, cost-effectiveness, and generalizability to low-resource settings	Not reported	Not reported	China, HK, the Netherlands	4 (MEDLINE, PubMed, PsycINFO, Cochrane)	2013–2019	18	Systematic review	Evidence of effectiveness of computerized CBT on anxiety and depression; interventions with an in-person element with a professional, peer, or parent were associated with greater effectiveness, adherence, and lower dropout than fully automatized or self-administered interventions.
Liverpool et al. [25]	Systematic review	(1) Identify modes of delivery used in children and young people’s digital mental health interventions (DHI), (2) explore influencing factors on usage and implementation, and (3) investigate ways in which the interventions have been evaluated and whether children and young people engage in DHIs	Not reported	Not reported	USA, Canada, Australia	4 (Cochrane Library, EMBASE, MEDLINE, PsycINFO)	2001–2018	83	Narrative synthesis	Six modes of delivery were identified: (1) websites, (2) games and computer-assisted programs, (3) apps, (4) robots and digital devices, (5) virtual reality, and (6) mobile text messaging. Two themes of intervention-specific (suitability, usability, and acceptability of the DHI) and person-specific (motivation, capability, opportunity) barriers and facilitators to CYP’s engagement emerged.
Murphy et al. [45]	Rapid scoping review	(1) Identifies populations in the APEC region that are at higher risk of the negative mental health impacts of COVID-19, (2) identifies needs and gaps in access to standard and e-mental health care among these populations, and (3) explores the potential of e-mental health to address these needs	Not reported	Not reported	USA, China, Philippines	3 (Medline, Embase, PsycINFO)	2019–2020	132	Narrative review	Evidence that e-mental healthcare can be a viable option for care delivery but that specific accessibility and acceptability factors must be considered.
Nicholas et al. [26]	Systematic review	Identify the types of self-management apps available for bipolar disorder and to assess their features and the quality of their content	NA	NA	NA	2 (Australian Google Play and iOs stores)	NA	82 apps	Systematic review	22% of apps addressed privacy and security by providing a privacy policy; 36% and 15% applied core psychoeducation principles and best-practice guidelines, respectively.
Oyebode et al. [46]	Thematic analysis	Evaluate mental health apps by identifying positive and negative factors affecting the effective delivery of mental health apps	88, 125 reviews	NA	NA	2 (Google Play, App Store)	NA	104 apps	Thematic analysis	Identified 21 negative themes (usability issues, content issues, ethical issues, customer support issues, billing issues) and 29 positive themes (aesthetically pleasing interface, app stability, customizability, high-quality content, content variation/diversity, personalized content, privacy and security, low-subscription cost).
Petrovic and Gaggioli [47]	Scoping review	Investigate and thematically synthesize the existing literature to understand the state of the art digital mental health tools for managing burden, stress, and overall adverse mental health outcomes for the informal caregivers of older adults	Not reported	Not reported	Not reported	3 (Summon search box, Cochrane Library, PubMed)	2016–2019	16	Thematic synthesis	Overall, digital mental health interventions contribute to reducing the caregiver burden, with a limitation in addressing specific coping skills or education regarding illnesses such as Alzheimer’s disease and dementia.
Simblett et al. [27]	Systematic review and meta-analysis	Examine the scope and efficacy of e-mental health interventions to treat symptoms of PTSD	3832 (eligible for meta-analysis)	Not reported	USA, The Netherlands, Australia	4 (Cochrane Library, MEDLINE, EMBASE, PsycINFO)	2001–2016	39	Meta-analysis	The results of the primary meta-analysis revealed a significant improvement in PTSD symptoms, in favor of the active intervention group, independent of the comparison condition, type of CBT-based intervention, and level of guidance provided.
Six et al. [28]	Systematic review and meta-analysis	Examine whether mental health apps with gamification elements differ in their effectiveness to reduce depressive symptoms compared to apps that lack these elements	8110	58.3% female, mean age = 35.6, SD = 7.9 years	Not reported	5 (PubMed, PsycINFO, Cochrane Clinical Trials Registry, Web of Science, PsyArXiv)	2011–2020	38	Meta-analysis	Results indicated a small to moderate effect size across all mental health apps in reducing depressive symptoms compared to controls; no difference in effectiveness between mental health apps with and without gamification elements.
Struthers et al. [29]	Systematic review	Examine the acceptability of e-mental health services for children, adolescents, and young adults and their parents and healthcare providers	Not reported	Mean age for all studies was <25 years	Australia, USA, UK	11 (PubMed/Medline, EMBASE, CINAHL, PsycINFO, Google Scholar, Science Citation Index/Science Citation Index Expanded, Web of Science, Prouest, www.clinicaltrials.gov, Cochrane Central Register of Controlled Trials, and Google)	1990–2012	24	Systematic review	Clients are generally satisfied with e-mental health and report positive experiences, although adherence and uptake can be challenges
Thach [48]	Qualitative analysis of user reviews	Examine which design factors of mental health apps are significant/essential/unnecessary to consumers, and which factors affect user adherence	1116 reviews	NA	NA	MHapps within the list reviewed by MH professionals published on website for Anxiety and Depression Association of America (ADAA)	User reviews posted from 2016 to 2017	Five CBT-based apps: Pacifica, Happify, MindShift, MoodToosl, Moodkit	Qualitative analysis	Users highly appreciate the ability to monitor and reflect on themselves, and to figure out what is going on in their mood. By contrast, key aspects of dissatisfaction include technical issues, lack of customer service, clear security measures, and privacy policy.
Thach [49]	Qualitative analysis of user reviews	In the context of cognitive behavioral therapy (CBT)-based mental health applications examine (1) who are intended users, (2) what they use these apps for, and (3) why they use it	1116 reviews	NA	NA	MHapps within the list reviewed by MH professionals published on website for Anxiety and Depression Association of America (ADAA)	User reviews posted from 2016 to 2017	Five CBT-based apps: Pacifica, Happify, MindShift, MoodToosl, Moodkit	Qualitative analysis	CBT-based apps are used to relax, track mood, practice mindfulness, self-care, or build healthy habits. Apps are used to understand one’s health, help to keep on progressing with health, to see correlation between causes and effects of one’s health problems, to conduct self-evaluation and self-reflection, to build good habits, and to provoke, reframe, and organize their thoughts.
Torous et al. [14]	Clinical review	Review current challenges surrounding user engagement with mental health smartphone apps	NA	NA	NA	NA	NA	NA	Narrative review	Identified that mental health smartphone apps are (1) not user-friendly, (2) are not designed in a user-centric manner, (3) do not respect privacy, (4) are seen as an untrustworthy source of mental health information, and (5) are unhelpful in emergency situations
Wies et al. [50]	Scoping review	Synthesize the growing literature on the benefits and ethical challenges of digital mental health for young people (children or adolescents) aged 0 to 25	Not reported	Not reported	Not reported	6 (PubMed, Scopus, World of Science, PsycINFO, IEEE Xplore, ACM Digital Library)	Up to 2020	26	Qualitative thematic synthesis	Identified diverse themes related to the opportunities (better understanding of mental health, patient empowerment and respect for autonomy, equality, increased accessibility, affordability, and availability of care) and ethical challenges (impact on patient-doctor relationship, insufficient validation of technological tools, risk of stigma, data security and privacy risks) of digital mental health technologies
Zhang et al. [30]	Systematic review	Examine the effectiveness, acceptability, usability, and safety of digital health technologies (DHTs) for people with mental health problems in China	3112	Mean age ranged from 4.7 to 47.4 years	Mainland China	7 (Medline, PsycINFO, EMBASE, Web of Science, CNKI, WANFANG, VIP)	2013–2021	39	Narrative synthesis	DHTs were acceptable and usable among Chinese people with mental health problems in general

**Table 3:** Target population of included reviews.

Included Reviews	Target Population
Alqahtani & Orji [32] Bakker et al. [33] Balcombe et al. [34] Carter et al. [36] Denecke et al. [37] Drissi et al. [38] Ellis et al. [39] Gould et al. [40] Hwang et al. [42] Kaveladze et al. [43] Lal & Adair [44] Lehtimaki et al. [24] Liverpool et al. [25] Murphy et al. [45] Oyebode et al. [46] Struthers et al. [29] Torous et al. [14] Wies et al. [50] Zhang et al. [30]	No restriction on the type of mental health condition
Binhadyan et al. [35]	ADHD
Borghouts et al. [18] Eisenstadt et al. [15] Firth et al. [19] Garrido et al. [20] Harith et al. [41] Lattie et al. [22] Leech et al. [23] Nicholas et al. [26] Petrovic & Gaggioli [47] Six et al. [28] Thach [48] Thach [49]	Anxiety/Depression/Stress/Well-being
Chan & Honey [9] Henson et al. [12]	Anxiety, Depression, Schizophrenia spectrum and psychotic disorders
Larsen et al. [21]	Suicide/Self-harm
Simblett et al. [27]	Post-traumatic stress disorder (PTSD)

## Data Availability

Not Applicable.

## References

[1] Chan S., Torous J., Hinton L., Yellowlees P. (2015). Towards a Framework for Evaluating Mobile Mental Health Apps. Telemed. E-Health.

[2] Clough B.A., Casey L.M. (2015). The Smart Therapist: A Look to the Future of Smartphones and MHealth Technologies in Psychotherapy. Prof. Psychol. Res. Pract..

[3] Kretzschmar K., Tyroll H., Pavarini G., Manzini A., Singh I., NeurOx Young People’s Advisory Group (2019). Can Your Phone Be Your Therapist? Young People’s Ethical Perspectives on the Use of Fully Automated Conversational Agents (Chatbots) in Mental Health Support. Biomed. Inform. Insights.

[4] Ha S.W., Kim J. (2020). Designing a Scalable, Accessible, and Effective Mobile App Based Solution for Common Mental Health Problems. Int. J. Hum. Comput. Interact..

[5] Marshall J.M., Dunstan D.A., Bartik W. (2020). Clinical or Gimmickal: The Use and Effectiveness of Mobile Mental Health Apps for Treating Anxiety and Depression. Aust. N. Z. J. Psychiatry.

[6] Seko Y., Kidd S., Wiljer D., McKenzie K. (2014). Youth Mental Health Interventions via Mobile Phones: A Scoping Review. Cyberpsychol. Behav. Soc. Netw..

[7] Price M., Yuen E.K., Goetter E.M., Herbert J.D., Forman E.M., Acierno R., Ruggiero K.J. (2014). MHealth: A Mechanism to Deliver More Accessible, More Effective Mental Health Care: MHealth Opportunities. Clin. Psychol. Psychother..

[8] Anderson J.K., Howarth E., Vainre M., Jones P.B., Humphrey A. (2017). A Scoping Literature Review of Service-Level Barriers for Access and Engagement with Mental Health Services for Children and Young People. Child. Youth Serv. Rev..

[9] Chan A.H.Y., Honey M.L.L. (2022). User Perceptions of Mobile Digital Apps for Mental Health: Acceptability and Usability—An Integrative Review. J. Psychiatr. Ment. Health Nurs..

[10] Cohen K.A., Stiles-Shields C., Winquist N., Lattie E.G. (2021). Traditional and Nontraditional Mental Healthcare Services: Usage and Preferences Among Adolescents and Younger Adults. J. Behav. Health Serv. Res..

[11] World Health Organization, Regional Office for South-East Asia (2017). Mental Health Status of Adolescents in South-East Asia: Evidence for Action.

[12] Henson P., Wisniewski H., Hollis C., Keshavan M., Torous J. (2019). Digital Mental Health Apps and the Therapeutic Alliance: Initial Review. BJPsych Open.

[13] Torous J., Chan S.R., Tan S.Y.-M., Behrens J., Mathew I., Conrad E.J., Hinton L., Yellowlees P., Keshavan M. (2014). Patient Smartphone Ownership and Interest in Mobile Apps to Monitor Symptoms of Mental Health Conditions: A Survey in Four Geographically Distinct Psychiatric Clinics. JMIR Ment. Health.

[14] Torous J., Nicholas J., Larsen M.E., Firth J., Christensen H. (2018). Clinical Review of User Engagement with Mental Health Smartphone Apps: Evidence, Theory and Improvements. Evid. Based Ment. Health.

[15] Eisenstadt M., Liverpool S., Infanti E., Ciuvat R.M., Carlsson C. (2021). Mobile Apps That Promote Emotion Regulation, Positive Mental Health, and Well-Being in the General Population: Systematic Review and Meta-Analysis. JMIR Ment. Health.

[16] Lindhiem O., Harris J.L., Moreno M.A., Radovic A. (2018). Apps for mental health. Technology and Adolescent Mental Health.

[17] Martin J. (2017). Joanna Briggs Institute 2017 Critical Appraisal Checklist for Systematic Reviews and Research Syntheses.

[18] Borghouts J., Eikey E., Mark G., De Leon C., Schueller S.M., Schneider M., Stadnick N., Zheng K., Mukamel D., Sorkin D.H. (2021). Barriers to and Facilitators of User Engagement with Digital Mental Health Interventions: Systematic Review. J. Med. Internet Res..

[19] Firth J., Torous J., Nicholas J., Carney R., Pratap A., Rosenbaum S., Sarris J. (2017). The Efficacy of Smartphone-Based Mental Health Interventions for Depressive Symptoms: A Meta-Analysis of Randomized Controlled Trials. World Psychiatry.

[20] Garrido S., Millington C., Cheers D., Boydell K., Schubert E., Meade T., Nguyen Q.V. (2019). What Works and What Doesn’t Work? A Systematic Review of Digital Mental Health Interventions for Depression and Anxiety in Young People. Front. Psychiatry.

[21] Larsen M.E., Nicholas J., Christensen H. (2016). A Systematic Assessment of Smartphone Tools for Suicide Prevention. PLoS ONE.

[22] Lattie E.G., Adkins E.C., Winquist N., Stiles-Shields C., Wafford Q.E., Graham A.K. (2019). Digital Mental Health Interventions for Depression, Anxiety, and Enhancement of Psychological Well-Being Among College Students: Systematic Review. J. Med. Internet Res..

[23] Leech T., Dorstyn D., Taylor A., Li W. (2021). Mental Health Apps for Adolescents and Young Adults: A Systematic Review of Randomised Controlled Trials. Child. Youth Serv. Rev..

[24] Lehtimaki S., Martic J., Wahl B., Foster K.T., Schwalbe N. (2021). Evidence on Digital Mental Health Interventions for Adolescents and Young People: Systematic Overview. JMIR Ment. Health.

[25] Liverpool S., Mota C.P., Sales C.M.D., Čuš A., Carletto S., Hancheva C., Sousa S., Cerón S.C., Moreno-Peral P., Pietrabissa G. (2020). Engaging Children and Young People in Digital Mental Health Interventions: Systematic Review of Modes of Delivery, Facilitators, and Barriers. J. Med. Internet Res..

[26] Nicholas J., Larsen M.E., Proudfoot J., Christensen H. (2015). Mobile Apps for Bipolar Disorder: A Systematic Review of Features and Content Quality. J. Med. Internet Res..

[27] Simblett S., Birch J., Matcham F., Yaguez L., Morris R. (2017). A Systematic Review and Meta-Analysis of e-Mental Health Interventions to Treat Symptoms of Posttraumatic Stress. JMIR Ment. Health.

[28] Six S.G., Byrne K.A., Tibbett T.P., Pericot-Valverde I. (2021). Examining the Effectiveness of Gamification in Mental Health Apps for Depression: Systematic Review and Meta-Analysis. JMIR Ment. Health.

[29] Struthers A., Charette C., Bapuji S.B., Winters S., Ye X., Metge C., Kreindler S., Raynard M., Lemaire J., Synyshyn M. (2015). The Acceptability of E-Mental Health Services for Children, Adolescents, and Young Adults: A Systematic Search and Review. Can. J. Community Ment. Health.

[30] Zhang X., Lewis S., Firth J., Chen X., Bucci S. (2021). Digital Mental Health in China: A Systematic Review. Psychol. Med..

[31] Aromataris E., Fernandez R.S., Godfrey C., Holly C., Khalil H., Tungpunkom P. (2014). Methodology for JBI Umbrella Reviews.

[32] Alqahtani F., Orji R. (2020). Insights from User Reviews to Improve Mental Health Apps. Health Inform. J..

[33] Bakker D., Kazantzis N., Rickwood D., Rickard N. (2016). Mental Health Smartphone Apps: Review and Evidence-Based Recommendations for Future Developments. JMIR Ment. Health.

[34] Balcombe L., Leo D.D. (2020). Psychological Screening and Tracking of Athletes and Digital Mental Health Solutions in a Hybrid Model of Care: Mini Review. JMIR Form. Res..

[35] Binhadyan B., Davey B., Wickramasinghe N. (2016). How e-mental health services benefit university students with ADHD: A literature review. arXiv.

[36] Carter H., Araya R., Anjur K., Deng D., Naslund J.A. (2021). The Emergence of Digital Mental Health in Low-Income and Middle-Income Countries: A Review of Recent Advances and Implications for the Treatment and Prevention of Mental Disorders. J. Psychiatr. Res..

[37] Denecke K., Schmid N., Nüssli S. (2022). Implementation of Cognitive Behavioral Therapy in e–Mental Health Apps: Literature Review. J. Med. Internet Res..

[38] Drissi N., Ouhbi S., Marques G., Díez I.D.L.T., Ghogho M., Janati Idrissi M.A. (2021). A Systematic Literature Review on E-Mental Health Solutions to Assist Health Care Workers During COVID-19. Telemed. E-Health.

[39] Ellis L.A., Meulenbroeks I., Churruca K., Pomare C., Hatem S., Harrison R., Zurynski Y., Braithwaite J. (2021). The Application of E-Mental Health in Response to COVID-19: Scoping Review and Bibliometric Analysis. JMIR Ment. Health.

[40] Gould C.E., Kok B.C., Ma V.K., Zapata A.M.L., Owen J.E., Kuhn E. (2019). Veterans Affairs and the Department of Defense Mental Health Apps: A Systematic Literature Review. Psychol. Serv..

[41] Harith S., Backhaus I., Mohbin N., Ngo H.T., Khoo S. (2022). Effectiveness of Digital Mental Health Interventions for University Students: An Umbrella Review. PeerJ.

[42] Hwang W.J., Ha J.S., Kim M.J. (2021). Research Trends on Mobile Mental Health Application for General Population: A Scoping Review. Int. J. Environ. Res. Public Health.

[43] Kaveladze B.T., Wasil A.R., Bunyi J.B., Ramirez V., Schueller S.M. (2022). User Experience, Engagement, and Popularity in Mental Health Apps: Secondary Analysis of App Analytics and Expert App Reviews. JMIR Hum. Factors.

[44] Lal S., Adair C.E. (2014). E-Mental Health: A Rapid Review of the Literature. Psychiatr. Serv..

[45] Murphy J.K., Khan A., Sun Q., Minas H., Hatcher S., Ng C.H., Withers M., Greenshaw A., Michalak E.E., Chakraborty P.A. (2021). Needs, Gaps and Opportunities for Standard and e-Mental Health Care among at-Risk Populations in the Asia Pacific in the Context of COVID-19: A Rapid Scoping Review. Int. J. Equity Health.

[46] Oyebode O., Alqahtani F., Orji R. (2020). Using Machine Learning and Thematic Analysis Methods to Evaluate Mental Health Apps Based on User Reviews. IEEE Access.

[47] Petrovic M., Gaggioli A. (2020). Digital Mental Health Tools for Caregivers of Older Adults—A Scoping Review. Front. Public Health.

[48] Thach K.S. A qualitative analysis of user reviews on mental health apps: Who used it? For what? And why? In Proceedings of the 2019 IEEE-RIVF International Conference on Computing and Communication Technologies (RIVF), Danang, Vietnam, 20–22 March 2019.

[49] Thach K.S. User’s perception on mental health applications: A qualitative analysis of user reviews. Proceedings of the 2018 5th NAFOSTED Conference on Information and Computer Science (NICS).

[50] Wies B., Landers C., Ienca M. (2021). Digital Mental Health for Young People: A Scoping Review of Ethical Promises and Challenges. Front. Digit. Health.

[51] Moher D., Liberati A., Tetzlaff J., Altman D.G. (2009). Preferred Reporting Items for Systematic Reviews and Meta-Analyses: The PRISMA Statement. BMJ.

[52] Khazaal Y., Favrod J., Sort A., Borgeat F., Bouchard S. (2018). Editorial: Computers and Games for Mental Health and Well-Being. Front. Psychiatry.

[53] Tremain H., McEnery C., Fletcher K., Murray G. (2020). The Therapeutic Alliance in Digital Mental Health Interventions for Serious Mental Illnesses: Narrative Review. JMIR Ment. Health.

[54] Bolinski F., Boumparis N., Kleiboer A., Cuijpers P., Ebert D.D., Riper H. (2020). The Effect of E-Mental Health Interventions on Academic Performance in University and College Students: A Meta-Analysis of Randomized Controlled Trials. Internet Interv..

[55] Burger F., Neerincx M.A., Brinkman W.-P. (2020). Technological State of the Art of Electronic Mental Health Interventions for Major Depressive Disorder: Systematic Literature Review. J. Med. Internet Res..

[56] Montague A.E., Varcin K.J., Simmons M.B., Parker A.G. (2015). Putting Technology into Youth Mental Health Practice: Young People’s Perspectives. SAGE Open.

[57] Simple Practice (2022). Top 10 Mental Health CPT® Codes Billed in 2021.

[58] Lua V.Y.Q., Majeed N.M., Hartanto A., Leung A.K.-Y. (2022). Help-Seeking Tendencies and Subjective Well-Being: A Cross-Cultural Comparison of the United States and Japan. Soc. Psychol. Q..

[59] Radovic A., Vona P.L., Santostefano A.M., Ciaravino S., Miller E., Stein B.D. (2016). Smartphone Applications for Mental Health. Cyberpsychol. Behav. Soc. Netw..

[60] Lau N., O’Daffer A., Colt S., Yi-Frazier J.P., Palermo T.M., McCauley E., Rosenberg A.R. (2020). Android and IPhone Mobile Apps for Psychosocial Wellness and Stress Management: Systematic Search in App Stores and Literature Review. JMIR mHealth uHealth.

[61] Torous J.B., Chan S.R., Gipson S.Y.-M.T., Kim J.W., Nguyen T.-Q., Luo J., Wang P. (2018). A Hierarchical Framework for Evaluation and Informed Decision Making Regarding Smartphone Apps for Clinical Care. Psychiatr. Serv..

[62] Smith A.C., Fowler L.A., Graham A.K., Jaworski B.K., Firebaugh M.-L., Monterubio G.E., Vázquez M.M., DePietro B., Sadeh-Sharvit S., Balantekin K.N. (2021). Digital Overload among College Students: Implications for Mental Health App Use. Soc. Sci..

[63] Hartanto A., Lua V.Y.Q., Quek F.Y.X., Yong J.C., Ng M.H.S. (2021). A Critical Review on the Moderating Role of Contextual Factors in the Associations between Video Gaming and Well-Being. Comput. Hum. Behav. Rep..

